# Supplementary material for: CRISPR-Cas9 targeted disruption of the yellow ortholog in the housefly identifies the brown body locus
Source: Sci Rep. 2017 Jul 4;7:4582. doi: 10.1038/s41598-017-04686-6 (PMC5496933; doi:10.1038/s41598-017-04686-6)
Supplement: Supplementary file 1 — Supplementary Info [file 41598_2017_4686_MOESM1_ESM.pdf]

Supplementary Materials for

CRISPR-Cas9 targeted disruption of the *yellow* ortholog in the housefly identifies the *brown body* locus

Svenia D. Heinze[1†], Tea Kohlbrenner[1†], Domenica Ippolito[1], Angela Meccariello[2], Alexa Burger[1], Christian Mosimann[1], Giuseppe Saccone[2], and Daniel Bopp[1*]

*correspondence to: daniel.bopp@imls.uzh.ch

Supplementary Figs. 1- 4

```
GCTGCCTACAAATTGCAAGAACGTTTCAATTGGAAACAATTGGATTTTGCTTTTCCCAAT  117  MdY
GCTGCTTACAAACTTCAGGAGCGATATAGTTGGAGCCAGCTGGACTTTGCTTTCCCGAAT  291  DmY

GAAAATCTAAAACAACAGGCTTTGGCCAGTGGAGATTATATTCCACAGAATGCTTTGCCC  177  MdY
ACCCGACTAAAGGACCAAGCTCTGGCTAGTGGAGATTATATTCCGCAAAATGCTCTACCT  351  DmY

GTAGGTGTTGAACA--TTGGCAGAATCCATCCACTATTTGTTACCATACCCCGTTGGAGA  235  MdY
GTTGGAGTCGAACACTTTGGCA------ATCGGTTATTCGTCACTGTTCCCCGCTGGCGT  405  DmY

GATGG---------------------------ATGGATCATAGCGTTACCGGTTCTCCT  267  MdY
GATGGGATTCCGGCCACTCTGACCTATATAAACATGGACCGCAGTTTGACGGGTTCACCG  465  DmY

GCATTGATACCCTATCCCGACTGGCGTTCAAATACAGCTGGAGATTGTGCAAACAGCATT  327  MdY
GAGCTAATTCCGTATCCAGATTGGCGCTCAAATACAGCTGGAGATTGCGCCAACAGTATT  525  DmY

ACCACAGCTTATCGTATTAAGGTTGATGAATGTGGTCGTTTGTGGGTTTTGGACACTGGC  387  MdY
ACCACTGCCTACCGCATTAAAGTGGATGAGTGTGGTCGGCTGTGGGTTTTGGACACTGGA  585  DmY

ACTTTGGGTATTGGCAACACCACCACCAACCCCTGTCCCTATGCCGTGAATGTTTTCGAT  447  MdY
ACCGTGGGCATCGGCAATACCACCACTAATCCGTGCCCCTATGCGGTAAATGTCTTTGAC  645  DmY

TTGGCCACACACACACGCATACGCCGCTATGAGCT--GAGGCCGGAGGACACGAATGCAA  505  MdY
TTGACCACGGATACGCGAATTCGGAGATACGAGCTACCTGGC--GTGGACACAAATCCAA  703  DmY

ATACTTTTATTGCCAACATTGCTGTGGACATTGGCAAGAGTTGTGATGATGCTTTTGCTT  565  MdY
ATACTTTCATAGCTAACATTGCCGTGGATATAGGCAAAAATTGCGATGATGCATATGCCT  763  DmY

ATTTCTCTGATGAGTTGGGTTATGGTTTGATTGCCTATTCATGGGAACAGAATAAGTCAT  625  MdY
ATTTTGCCGATGAATTGGGATACGGCTTGATTGCTTACTCCTGGGAACTGAACAAGTCCT  823  DmY

GGCGTTTCTCGGGACATTCGTACTTCTTCCCCGATCCCTTGAGAGGTGATTACAACATTG  685  MdY
GGAGATTCTCGGCACATTCGTATTTTTTCCCCGATCCATTGAGGGGCGATTTCAATGTCG  883  DmY

CCGGTCTCAATTTCCAGTGGGGTGAGGAGGGCATCTTTGGCATGGCTTTGTCGCCCATTC  745  MdY
CTGGTATTAACTTCCAATGGGGCGAGGAGGGTATATTTGGTATGTCCCTTTCGCCCATTC  943  DmY

GTTCGGATGGTTATCGCACGATGTATTTCAGCCCCCTGGCCAGTCATAGGCAATTTGCTG  805  MdY
GATCGGATGGTTATCGTACCCTGTACTTTAGTCCGTTAGCAAGTCATCGACAATTTGCCG  1003 DmY

TATCCACTCGGATTTTGCGTGATGAAACTCGTGTTGAGGATAGCTATCATGACTTTATTG  865  MdY
TATCCACGAGGATTTTGAGGGATGAAACCAGGACGGAAGATAGCTATCATGACTTTGTTG  1063 DmY

CTTTGGATGAACGTGGACCCAATTCTCACACCACTGCCCGTGTTATGAGCGAAGATGGTG  925  MdY
CCTTAGATGAACGGGGTCCAAACTCCCATACCACTTCACGTGTGATGAGCGATGATGGAA  1123 DmY

TTGAGTTGTTCAATTTAATCGATCAAAATTCTGTGGGTTGTTGGCATTCGTCCATGCCCT  985  MdY
TTGAGCTGTTCAATTTAATAGATCAAAATGCAGTGGGTTGCTGGCACTCATCAATGCCGT  1183 DmY

ACTCACCCCAGTTCCATGGTATTGTGGATCGTGATGATGTGGGTCTGGTGTTCCCCGCTG  1045 MdY
ACTCACCGCAATTTCATGGCATTGTGGATCGCGATGACGTTGGCTTAGTTTTTCCGGCCG  1243 DmY

ATGTTAAAATCGATGAAAACAAAGATGTTTGGGTACTCTCAGATCGTATGCCGGTGTTTT  1105 MdY
ATGTGAAAATTGATGAGAACAAAAACGTTTGGGTTCTATCCGATAGGATGCCCGTTTTCT  1303 DmY

TGCTTTCCGAATTGGATTACAATGATGTGAACTTCCGTATCTATACCGCTCCCCTGAGCA  1165 MdY
TGCTGTCTGACTTGGATTATTCAGATACTAATTTCCGAATTTACACGGCTCCCTTGGCCA  1363 DmY

CGCTCATTGAGGGCACTGTCTGCGAT  1191  MdY
CTTTAATTGAGAATACTGTGTGTGAT  1389  DmY
```

Supplementary Figure 1. Nucleotide alignment of Drosophila *yellow* (*DmY*) and Musca *yellow* (*MdY*). The sequences used for alignment are XM_011297481.1 for Musca domestica *yellow* mRNA and NM_057444.3 for Drosophila melanogaster *yellow* mRNA. Overall identity is 74%

a

```
Musca       ----MKCPLVGLLTFVLCIQFGNAAYKLQERFNWKQLDFAFPNENLKQQALASGDYIPQN
Stomoxys    ----MKCFLVGLLTFVLCVQFGYATYKLQERFNWKQLDFAFPNENLKQQALASGDYIPQN
Drosophila  MFQDKGWILVTLIT---LVTPSWAAYKLQERYSWSQLDFAFPNTRLKDQALASGDYIPQN
Bactrocera  MQANLRLTLRNLVAWLCLVAHAQATYKLQERYSWTELDFAFPNQGLKQQALASGDYIPQN
Ceratitis   MFANSHLSLRSIVACLCLVAHAQAAYKLQERYSWTQMDFAFPNPGLKQQALASGDYIPQN
                    *  :::    :  . *:******:.*.::******  **:************

Musca       ALPVGVEHWQNRLFVTIPRWRDGIPATLTYINMDHSVTGSPALIPYPDWRSNTAGDCANS
Stomoxys    ALPVGVEHWQNRLFVTVPRWRDGIPATLTYINMEHSVTGSPALIPYPDWRSNTAGDCANG
Drosophila  ALPVGVEHFGNRLFVTVPRWRDGIPATLTYINMDRSLTGSPELIPYPDWRSNTAGDCANS
Bactrocera  ALPVGVEHWGNRLFVTVPRWRDGIPATLTYINMDHSVTGSPELIPYPDWRSNTAGDCANS
Ceratitis   ALPVGMEHWGNRLFVTVPRWRDGIPATLTYINMDHSATGSPELIPYPDWRSNTAGDCANS
            *****:**: ******:****************:.* **** *****************.

Musca       ITTAYRIKVDECGRLWVLDTGTLGIGNTTTNPCPYAVNVFDLATHTRIRRYELRPEDTNA
Stomoxys    ITTAYRIKVDECGRLWVLDTGTIGIGNTTTNPCPYAVNVFDLATHTRIRRYELNPEDTNA
Drosophila  ITTAYRIKVDECGRLWVLDTGTVGIGNTTTNPCPYAVNVFDLTTDTRIRRYELPGVDTNP
Bactrocera  ITTAYRIKADECGRLWVLDTGTVGIGNTTTNPCPYAVNVFDLQTNTRIRRYVLRADDTNA
Ceratitis   ITTAYRIRADECGRLWVLDTGTVGIGNTTTNPCPYAVNVYDLQTNTRIRHYELRAEDTNA
            *******..*************:****************:** * ****.* *  ***.*

Musca       NTFIANIAVDIGKSCDDAFAYFSDELGYGLIAYSWEQNKSWRFSGHSYFFPDPLRGDYNI
Stomoxys    NTFIANIAVDIGKSCDDAFAYFSDELGYGLISYSWEQNKSWRFSGHSYFFPDPLRGDYNI
Drosophila  NTFIANIAVDIGKNCDDAYAYFADELGYGLIAYSWELNKSWRFSAHSYFFPDPLRGDFNV
Bactrocera  NTFIANVAVDIGKSCDDAFAYFSDELGYGLVVYSWEQNKSWRFSAHSYFFPDPLRGDFNI
Ceratitis   NTFVANIAVDIGKSCDDAFAYFSDELGYGLIVYSWEQNKSWRFSAHSYFFPDPLRGDFNI
            ***:**:******.****:***:*******: **** *******.************:*:

Musca       AGLNFQWGEEGIFGMALSPIRSDGYRTMYFSPLASHRQFAVSTRILRDETRVEDSYHDFI
Stomoxys    AGLNFQWGEEGIFGMALSPLRSDGFRTMYFSPLASHRQFAVSTRILRDESRVEDSYHDFI
Drosophila  AGINFQWGEEGIFGMSLSPIRSDGYRTLYFSPLASHRQFAVSTRILRDETRTEDSYHDFV
Bactrocera  AGLNFQWGEEGIFGMSLSPIRSDGYRTMFFSPLASHRQFAVSTRILRDESRVEDSFHDFV
Ceratitis   AGLNFQWGEEGIFGMALSPIRSDGYRTMFFSPLASHRQFAVSTRILRDESRVEDSYHDFV
            **:************:***:****:**::********************:*.***:***:

Musca       ALDERGPNSHTTARVMSEDGVELFNLIDQNSVGCWHSSMPYSPQFHGIADRGDVGLVFPA
Stomoxys    ALDERGPNSHTTARVMSDDGVELFDLIDQNAVGCWHSSMPYSPQFHGIVDRDDVGLVFPA
Drosophila  ALDERGPNSHTTSRVMSDDGIELFNLIDQNAVGCWHSSMPYSPQFHGIVDRDDVGLVFPA
Bactrocera  VLDERGPNSHTTSRVMSDDGIELFNLIDQNAVGCWHSSMPYQPQFHGVVDRDDVGLVFPA
Ceratitis   ALDERGPNSHTTSRVMSDDGVELFNLIDQNAVGCWHSSMPYTSQFHGVVDRDDVGLVFPA
            .***********:****:**:***:*****:********** .****:.**.********

Musca       DVKIDESKDVWVLSDRMPVFLLSKLDYNDVNFRIYTAPLSTLIEGTVCDQRSNV-YGPHN
Stomoxys    DVKIDENKNVWVLSDRMPVFLLSELDYNDINFRIYTAPLSTLIEGTVCDQRTSSVYGPPN
Drosophila  DVKIDENKNVWVLSDRMPVFLLSDLDYSDTNFRIYTAPLATLIENTVCDLRNNA-YGPPN
Bactrocera  DVKIDENKNVWVLSDRMPVFLISDLDYNDVNFRIYTAPLSALIDNTVCDIRNNA-YGPSN
Ceratitis   DIKIDENKNVWVLSDRMPVFLITELDYNDVNFRIYTAPLSSLIENTVCDIRNSA-YGPSN
            *:****.*:************::.***.* *********::**:.**** *..  *** *

Musca       SVAA---------------------------------VKPLHPIYPKTYLNPT-AKPS--
Stomoxys    SVAA---------------------------------VKPQFPLYTKQYLPPAPAKPTII
Drosophila  TVSI----------------------------PKQAVLPMGPPLYTKQYRPVLPQKPQTS
Bactrocera  AVGSTSFYATPTSTNSVFGTQLGFVGNKVYGPPNPVGLPKSQTLFTKQYLPPLSIKPS-I
Ceratitis   SVGSTSFYGTPTSTNNIFGPQLNLGGNKVYGPPNPVSVPKSQTRFNKPYLPPLPTKPT-L
            :*.                                  :    . : * *      **

Musca       --YVA--IPSSRPSYLPP-----YS---------GSQRPNVPNAFLYNQQHNALTYDAAN
Stomoxys    QHHVP--ISSARPTYLPP-----YS---------GSQRLNVPSAFVYN-QHNGLSYEASN
Drosophila  WASSP--PPPSRT-YLPANSGNVVSSISVSTNSVGPAGVEVPKAYIFN-QHNGINYETS-
Bactrocera  QADVPRVAPPSRN-YLPPLMGTGYS---------TTQRSDPAKAYVFN---NGLSYETGI
Ceratitis   QADIPRVAPPSRN-YLPPLMGTGYS---------TTQRSDAAKAYVFN---NGLSYEAGV
                .   ..:*  ***.      *          .   : ..*:::*   *.:.*::.

Musca       -GPHLFPAIAQIQQIQHPAPAARE--GLGSYATSRSVPWWQQHH--
Stomoxys    -GPHLFPAIAHIQQIQHTAQPE----GLGSYATSRNTPWWKRQ---
Drosophila  -GPHLFP-------THQPAQPGGQDGGLKTYVNARQSGWWHHQHQG
Bactrocera  GGPHLFP-------LHSE--------GLKNYVTTRNSGWWTHH---
Ceratitis   GGPHLFP-------LHSE--------GLRNYVSARTSGWWLEH---
             ******        :          ** .*..:*   **  :
```

b

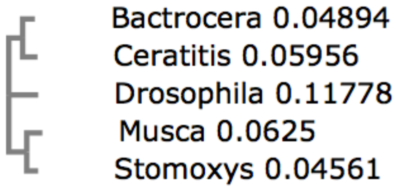

Supplementary Figure 2. Amino acid sequence alignments of *yellow* orthologs found in higher dipterans
a) Clustal Omega alignment was performed with the following GenBank entries: *Drosophila melanogaster* yellow protein ID NP_476792.1, *Stomoxys calcitrans* yellow protein ID XP_013112765.1, *Bactrocera oleae* yellow protein ID XP_014099614.1, *Ceratitis capitata* yellow protein ID XP_004521097.1 **b)**The resulting Neighbour-joining tree with real branch length is depicted

```
MdYa1/2  ATGAAGTGTCTTCTAGTTGGCCTATTGACATTTGTGTTGTGCATCCATTTCGGCAATGCTGCCTACAAATTGCAAGAACGTTTCAATTGGAAACAATTGGATTTTGCTTTTCCCAATGAA
MdYb     ATGAAGTGTCTTCTAGTTGGCCTATTGACATTTGTGTTGTGCATCCATTTCGGCAATGCTGCCTACAAATTGCAAGAACGTTTCAATTGGAAACAATTGGATTTTGCTTTTCCCAATGAA
MdY      ATGAAGTGTCTTCTAGTTGGCCTATTGACATTTGTGTTGTGCATCCATTTCGGCAATGCTGCCTACAAATTGCAAGAACGTTTCAATTGGAAACAATTGGATTTTGCTTTTCCCAATGAA
         ************************************************************************************************************************

MdYa1/2  AATCTAAAACAACAGGCCTTGGCCAGTGGAGATTATATTCCGCAGAATGCTTTACCCGTGGGTGTTGAACATTGGCAGAATCCATCCACTATTTGTTACCATACCCCGTTGGAGAGATGG
MdYb     AATCTAAAACAACAGGCCTTGGCCAGTGGAGATTATATTCCGCAGAATGCTTTACCCGTGGGTGTTGAACATTGACAGA----ATCGTCTATTTGTTACCATACCCCGTTGGAGAGATGG
MdY      AATCTAAAACAACAGGCCTTGGCCAGTGGAGATTATATTCCGCAGAATGCTTTACCCGTGGGTGTTGAACATTGGCAGA----ATCGTCTATTTGTTACCATACCCCGTTGGAGAGATGG
         ************************************************************************** ****    *************************************

MdYa1/2  TATACCAGCCACATTGACTTACATTAATATGGATCATAGCGTTACCGGTTCTCCTGCATTGATACCCTATCCCGATTGGCGTTCAAATACAGCTGGAGATTGTGCAAACAGCATTACCAC
MdYb     TATACCAGCCACATTGACTTACATTAATATGGATCATAGCGTTACCGGTTCTCCTGCATTGATACCCTATCCCGATTGGCGTTCAAATACAGCTGGAGATTGTGCAAACAGCATTACCAC
MdY      TATACCAGCCACATTGACTTACATTAATATGGATCATAGCGTTACCGGTTCTCCTGCATTGATACCCTATCCCGATTGGCGTTCAAATACAGCTGGAGATTGTGCAAACAGCATTACCAC
         ************************************************************************************************************************

MdYa1/2  AGCTTATCGTATTAAGGTTGATGAATGTGGTCGTTTGTGGGTTTTAGACACTGGCACCTTGGGTATTGGCAACACCACCACCAACCCCTGTCCCTATGCCGTGAATGTTTTCGATTTGGC
MdYb     AGCTTATCGTATTAAGGTTGATGAATGTGGTCGTTTGTGGGTTTTAGACACTGGCACCTTGGGTATTGGCAACACCACCACCAACCCCTGTCCCTATGCCGTGAATGTTTTCGATTTGGC
MdY      AGCTTATCGTATTAAGGTTGATGAATGTGGTCGTTTGTGGGTTTTAGACACTGGCACCTTGGGTATTGGCAACACCACCACCAACCCCTGTCCCTATGCCGTGAATGTTTTCGATTTGGC
         ************************************************************************************************************************

MdYa1/2  CACACACACACGCATACGCCGCTATGAGTTGAGACCGGAGGACACGAATGCAAATACTTTTATTGCCAACATTGCTGTGGACATCGGCAAGAGTTGTGATGATGCTTTCGCTTATTTCTC
MdYb     CACACACACACGCATACGCCGCTATGAGTTGAGACCGGAGGACACGAATGCAAATACTTTTATTGCCAACATTGCTGTGGACATCGGCAAGAGTTGTGATGATGCTTTCGCTTATTTCTC
MdY      CACACACACACGCATACGCCGCTATGAGTTGAGACCGGAGGACACGAATGCAAATACTTTTATTGCCAACATTGCTGTGGACATCGGCAAGAGTTGTGATGATGCTTTCGCTTATTTCTC
         ************************************************************************************************************************

MdYa1/2  TGATGAGTTGGGTTATGGTTTGATTGCCTATTCATGGGAACAGAATAAGTCATGGCGTTTCTCGGGACATTCGTACTTCTTCCCCGATCCCTTGAGAGGTGATTACAACATTGCCGGTCT
MdYb     TGATGAGTTGGGTTATGGTTTGATTGCCTATTCATGGGAACAGAATAAGTCATGGCGTTTCTCGGGACATTCGTACTTCTTCCCCGATCCCTTGAGAGGTGATTACAACATTGCCGGTCT
MdY      TGATGAGTTGGGTTATGGTTTGATTGCCTATTCATGGGAACAGAATAAGTCATGGCGTTTCTCGGGACATTCGTACTTCTTCCCCGATCCCTTGAGAGGTGATTACAACATTGCCGGTCT
         ************************************************************************************************************************

MdYa1/2  CAACTTCCAGTGGGGTGAAGAGGGCATATTCGGCATGGCTTTGTCGCCCATTCGTTCGGATGGTTATCGCACCATGTATTTCAGCCCCCTGGCCAGTCATAGGCAATTTGCTGTATCCAC
MdYb     CAACTTCCAGTGGGGTGAAGAGGGCATATTCGGCATGGCTTTGTCGCCCATTCGTTCGGATGGTTATCGCACCATGTATTTCAGCCCCCTGGCCAGTCATAGGCAATTTGCTGTATCCAC
MdY      CAACTTCCAGTGGGGTGAAGAGGGCATATTCGGCATGGCTTTGTCGCCCATTCGTTCGGATGGTTATCGCACCATGTATTTCAGCCCCCTGGCCAGTCATAGGCAATTTGCTGTATCCAC
         ************************************************************************************************************************

MdYa1/2  TAGGATTTTGCGTGATGAAACTCGTGTTGAGGATAGCTATCATGACTTCCTTGCTTTGGATGAACGTGGACCCAATTCTCACACCACTGCCCGTGTTATGAGCGAAGATGGTGTTGAGTT
MdYb     TAGGATTTTGCGTGATGAAACTCGTGTTGAGGATAGCTATCATGACTTCCTTGCTTTGGATGAACGTGGACCCAATTCTCACACCACTGCCCGTGTTATGAGCGAAGATGGTGTTGAGTT
MdY      TAGGATTTTGCGTGATGAAACTCGTGTTGAGGATAGCTATCATGACTTCCTTGCTTTGGATGAACGTGGACCCAATTCTCACACCACTGCCCGTGTTATGAGCGAAGATGGTGTTGAGTT
         ************************************************************************************************************************

MdYa1/2  GTTCAATTTAATCGATCAGAATGCGGTGGGTTGTTGGCACTCATCCATGCCCTACTCACCCCAGTTCCATGGTATTGTGGATCGTGATGATGTGGGTCTGGTGTTCCCTGCTGATGTTAA
MdYb     GTTCAATTTAATCGATCAGAATGCGGTGGGTTGTTGGCACTCATCCATGCCCTACTCACCCCAGTTCCATGGTATTGTGGATCGTGATGATGTGGGTCTGGTGTTCCCTGCTGATGTTAA
MdY      GTTCAATTTAATCGATCAGAATGCGGTGGGTTGTTGGCACTCATCCATGCCCTACTCACCCCAGTTCCATGGTATTGTGGATCGTGATGATGTGGGTCTGGTGTTCCCTGCTGATGTTAA
         ************************************************************************************************************************

MdYa1/2  AATAGATGAAAACAAAGATGTTTGGGTACTCTCAGATCGTATGCCGGTGTTTTTGCTTTCCGAATTGGATTACAATGATGTGAACTTCCGTATCTATACCGCTCCCCTGAGCACGCTCAT
MdYb     AATAGATGAAAACAAAGATGTTTGGGTACTCTCAGATCGTATGCCGGTGTTTTTGCTTTCCGAATTGGATTACAATGATGTGAACTTCCGTATCTATACCGCTCCCCTGAGCACGCTCAT
MdY      AATAGATGAAAACAAAGATGTTTGGGTACTCTCAGATCGTATGCCGGTGTTTTTGCTTTCCGAATTGGATTACAATGATGTGAACTTCCGTATCTATACCGCTCCCCTGAGCACGCTCAT
         ************************************************************************************************************************

MdYa1/2  TGAGGGCACTGTCTGCGATCAACGCAGCAATGTGTATGGCCCCCACAACTCAGTTGCCGCTGTAAAGCCCCTACATCCCATCTATCCCAAGACGTACTTAAATCCCACAGCCAAGCCATC
MdYb     TGAGGGCACTGTCTGCGATCAACGCAGCAATGTGTATGGCCCCCACAACTCAGTTGCCGCTGTAAAGCCCCTACATCCCATCTATCCCAAGACGTACTTAAATCCCACAGCCAAGCCATC
MdY      TGAGGGCACTGTCTGCGATCAACGCAGCAATGTGTATGGCCCCCACAACTCAGTTGCCGCTGTAAAGCCCCTACATCCCATCTATCCCAAGACGTACTTAAATCCCACAGCCAAGCCATC
         ************************************************************************************************************************

MdYa1/2  CTACGTAGCAATTCCCTCATCACGTCCCTCATATTTGCCACCCTACAGTGGCTCACAGCGCCCGAATGTTCCTAATGCCTTTTTGTACAATCAACAACACAATGCCCTTACCTATGATGC
MdYb     CTACGTAGCAATTCCCTCATCACGTCCCTCATATTTGCCACCCTACAGTGGCTCACAGCGCCCGAATGTTCCTAATGCCTTTTTGTACAATCAACAACACAATGCCCTTACCTATGATGC
MdY      CTACGTAGCAATTCCCTCATCACGTCCCTCATATTTGCCACCCTACAGTGGCTCACAGCGCCCGAATGTTCCTAATGCCTTTTTGTACAATCAACAACACAATGCCCTTACCTATGATGC
         ************************************************************************************************************************

MdYa1/2  CGCGAATGGACCTCATCTATTCCCGGCGATAGCACAAATCCAACAGATTCAACATCCCGCACCAGCTGCCCGTGAAGGTCTGGGTAGTTATGCCACATCACGTTCCGTTCCCTGGTGGCA
MdYb     CGCGAATGGACCTCATCTATTCCCGGCGATAGCACAAATCCAACAGATTCAACATCCCGCACCAGCTGCCCGTGAAGGTCTGGGTAGTTATGCCACATCACGTTCCGTTCCCTGGTGGCA
MdY      CGCGAATGGACCTCATCTATTCCCGGCGATAGCACAAATCCAACAGATTCAACATCCCGCACCAGCTGCCCGTGAAGGTCTGGGTAGTTATGCCACATCACGTTCCGTTCCCTGGTGGCA
         ************************************************************ *************************************************************

MdYa1/2  GCAACATCACTAG
MdYb     GCAACATCACTAG
MdY      GCAACATCACTAG
         *************
```

Supplementary Figure 3. Nucleotide alignment of 2 different nonsense *MdY* alleles found in the aabys strain and in the *M*^{III} strain

The nucleotides substitutions are labeled in red

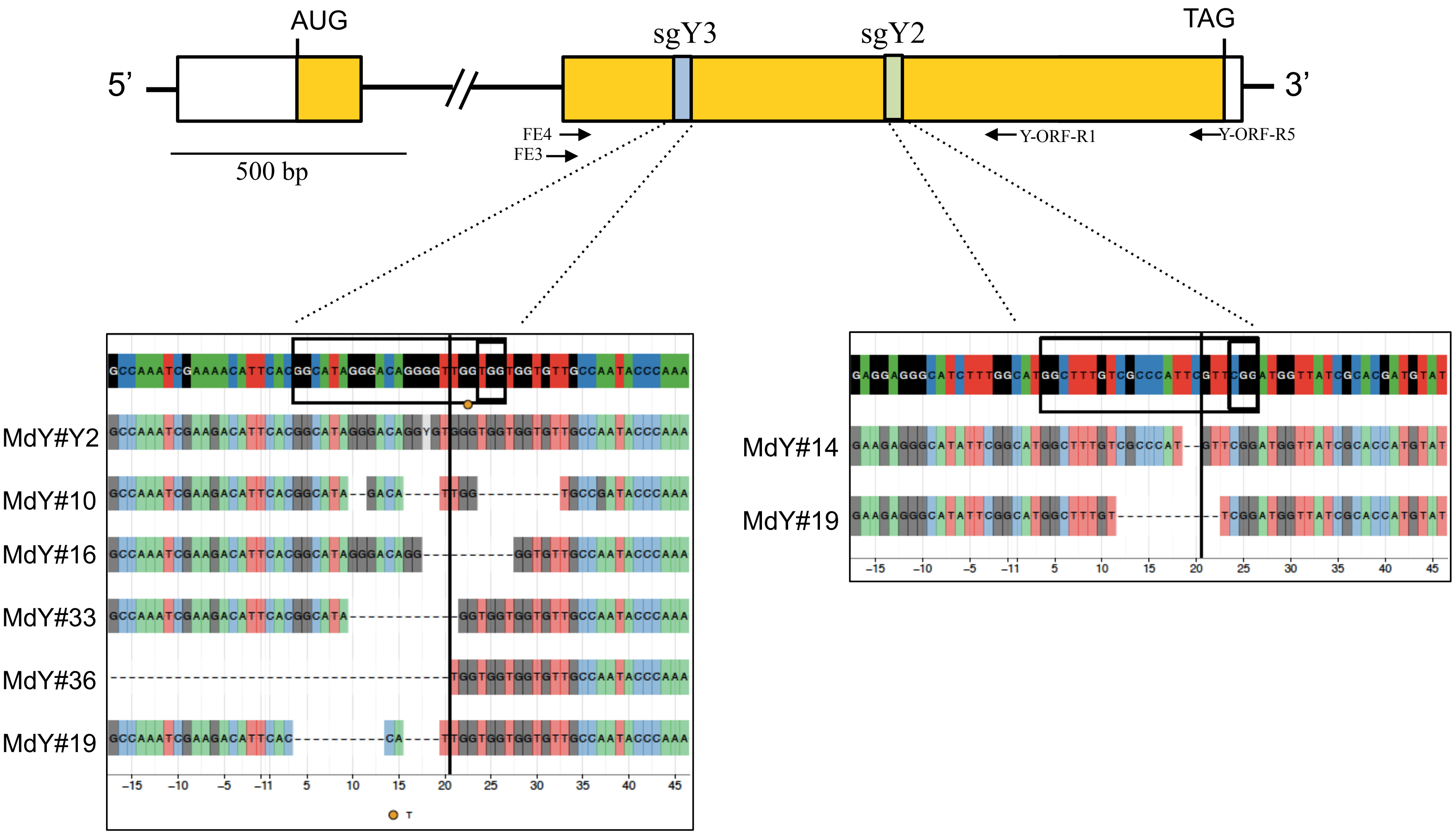

Supplementary Figure 4. Compilation of lesions generated in targeted sites.

Primers used for amplification and subcloning of fragments that were sequences are indicated with small arrows. CrispRVariants and the generation of panel plots was performed from primary sequencing data as described in [17] [18]
